# Streptococcus anginosus Lung Abscess With Complicated Parapneumonic Empyema

**DOI:** 10.7759/cureus.37506

**Published:** 2023-04-12

**Authors:** Laura M Gonzalez, Lutfor Nessa, Raghavendra Sanivarapu, Barath Rangaswamy, Laura Rojo

**Affiliations:** 1 Internal Medicine, Permian Basin Campus, Texas Tech University Health Sciences Center, Odessa, USA; 2 Pulmonary and Critical Care Medicine, Permian Basin Campus, Texas Tech University Health Sciences Center, Midland, USA; 3 General Medicine, Universidad Pontificia Bolivariana, Medellin, COL

**Keywords:** complicated community acquired pneumonia, parapneumonic effusion, • lung abscess, thoracic empyema, streptococcus anginosus group

## Abstract

A 55-year-old female with hypertension presented to our facility with complicated pneumonia. She complained of progressively worsening shortness of breath and pleuritic chest pain. She was in her usual state of health except for an upper respiratory infection treated with oral antibiotics a month prior. At the presentation, she was febrile, tachycardic, and hypoxic on room air. A chest computed tomography (CT) showed near-complete opacification of the right lung, a cavitation with the fluid level in the right middle lobe, and moderate-to-large effusion. Broad-spectrum antibiotics were started. Sputum culture was later positive for methicillin-resistant *Staphylococcus aureus*, which prompted antibiotic de-escalation to vancomycin. A chest tube was placed into the right pleural space draining 700 mL of exudative fluid, which cultures grew *Streptococcus anginosus *group (SAG) bacteria. Due to persistent respiratory distress and residual effusion, right thoracotomy and decortication were performed. A right upper lobe abscess ruptured into the pleural space was noted during the procedure. Pathology revealed necrotic tissue, and the microbiological workup was negative. The patient clinically improved postoperatively and was discharged home with oral Linezolid.

## Introduction

*Streptococcus anginosus* group (SAG) species colonize the oropharyngeal cavity, upper respiratory tract, gastrointestinal tract, and urogenital tract mucosa [1]. Recent reports have shown that this group is thought to account for 2% to 12% of community-acquired pneumonia, particularly among elderly individuals [1,2]. Its members have recently been linked with various complicated infections, mostly involving the formation of abscesses in the thoracic cavity, brain, and soft tissues [3]. Empyema is a bacterial infection of the pleural space that results in abscess formation. It is a serious infection, and its incidence has been increasing worldwide [4]. We present a case of lung abscess caused by SAG infection complicated with empyema requiring surgical intervention.

## Case presentation

**Figure 1:**
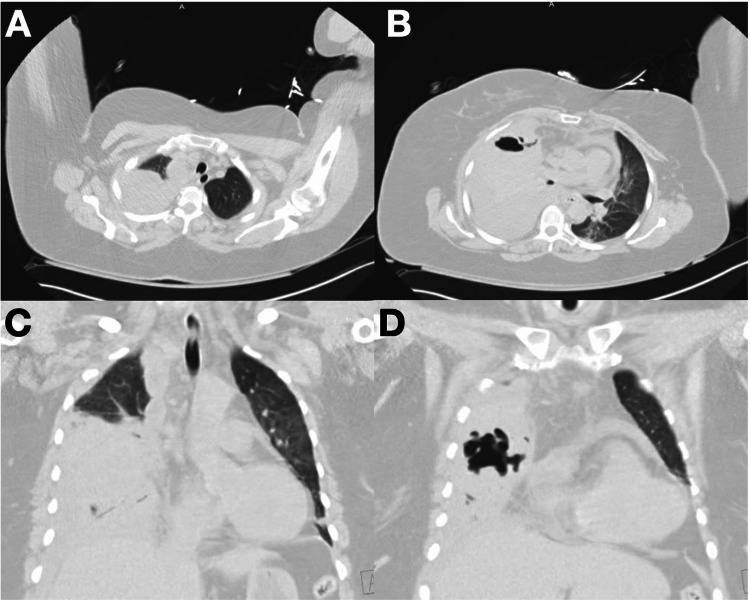
Computed tomography of the chest without contrast performed on the day of admission. (A and B) Computed tomography of the chest without contrast axial view on the day of admission demonstrated near-complete opacification of the right lung and a small amount of aerated parenchyma in the right upper lobe. (C and D) Computed tomography of the chest coronal view without contrast on the day of admission. There is a cavitation with a probable fluid level in the right middle lobe. Also showing moderate-to-large, low-density right pleural effusion.

**Figure 2:**
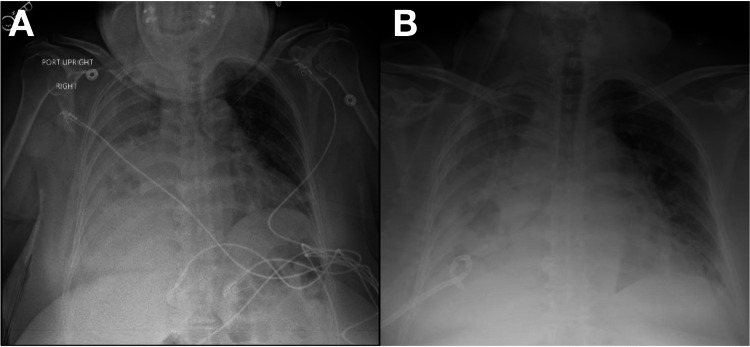
X-ray of the chest before and after right intrapleural chest tube placement. (A) AP X-ray of the chest frontal view on the day of admission demonstrates large right-sided pleural and parenchymal opacities, consistent with pleural fluid and associated volume loss/pneumonia. (B) AP X-ray of the chest frontal view on the second day of admission and interval placement of right pleural drain shows partial improvement in expansion of the right lung, which is still significantly hypoinflated. AP, anteroposterior

A 55-year-old female with a history of hypertension and no history of intravenous drug use presented as a transfer from an outside facility due to complicated pneumonia. She was treated for an upper respiratory tract infection with oral ciprofloxacin a month prior. However, she became progressively short of breath for about two weeks and presented with right pleuritic chest pain, which started two days before the admission. At presentation, she was febrile (101.2 °F), tachycardic (118 bpm), and hypoxic (87% on room air). The patient was placed on noninvasive positive pressure ventilation and was admitted to ICU. A physical exam revealed diminished breath sounds in the right lung base, with minimal wheezing scattered throughout both lungs. Initial laboratories showed leukocytosis (17 × 10^3^ mcL^-1^) with 47% bands, normochromic normocytic anemia (hemoglobin 11.5 g/dL), and thrombocytosis 548 × 10^3 ^mcL^-1^. Procalcitonin was elevated at 2.43 ng/mL, and C-reactive protein was 17.3 mg/dL. Computed tomography (CT) of the chest demonstrated near-complete opacification of the right lung, cavitation with a fluid level in the right middle lobe, and moderate-to-large effusion (Figure 1). Empiric broad-spectrum antibiotic coverage was started with intravenous vancomycin, azithromycin, and piperacillin-tazobactam. Thoracentesis with chest tube insertion in the right pleural space was performed the next day, with 700 mL of serosanguineous fluid drainage in the first 24 hours (Figure 2). Fluid analysis showed WBC 175 cells/mcL, lactate dehydrogenase (LDH) 2,240 units/L, glucose <2 mg/dL, pH 8, pertinent for exudative pleural effusion. Initial sputum cultures resulted positive for methicillin-resistant *Staphylococcus aureus*, prompting antibiotic de-escalation to vancomycin, and blood cultures from the day of admission did not grow any organism. Right pleural fluid culture grew SAG bacteria, confirming parapneumonic empyema. 

**Figure 3:**
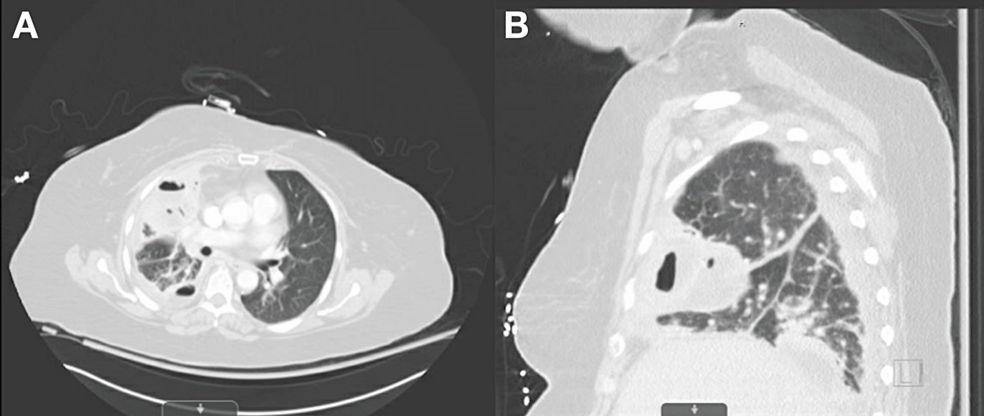
Computed tomography of the chest with intravenous contrast. Computed tomography with intravenous contrast after two rounds of intrapleural thrombolysis. (A) Axial and (B) coronal views showed a loculated fluid collection in the right oblique fissure between the right middle lobe and the right lower lobe. Additional smaller pleural loculations are redemonstrated abutting the right anterior chest wall with a small loculated hydropneumothorax.

Infectious disease and pulmonology services were consulted. Vancomycin was switched to clindamycin and ceftaroline. The patient still had residual pleural effusion and respiratory distress. Intrapleural fibrinolysis with a combination of tissue plasminogen activator (tPA) and dornase alfa (DNase) was performed twice through the chest tube. Each time, 4 mg of tPA in 50 mL of normal saline was left to dwell for four hours, followed by dornase 5 mg in 30 mL of normal saline to dwell for one hour. The patient continued to be febrile with leukocytosis and showed no improvement in oxygen requirements. Repeated CT chest on day 10 of admission showed multiloculated right-sided empyema or evolving fibrothorax, with improved right upper, middle, and lower lobe consolidations (Figure 3). Cardiothoracic surgery was consulted, and the patient underwent a right thoracotomy and decortication. A thick visceral pleural peel covering the entire right lung was found. A right upper lobe abscess rupture into the pleural space was noted, which was later found to be necrotic and negative for microbiology. The patient clinically improved postoperatively and was discharged home with oral linezolid to complete 30 days. She was seen one month later at the infectious disease clinic after completion of antibiotics. She was asymptomatic at that time.

## Discussion

Approximately 4% of all community-acquired pneumonia are necrotizing, although studies evaluating the incidence have found it to increase during the past 20 years. Common presenting symptoms include fever, tachypnea, and cough. More than 85% of those afflicted also develop complications such as parapneumonic effusions, empyemas, or fistulae [5]. Several pathogens are implicated in necrotizing pneumonia, with *S. aureus* being the most common in adults.

Pleural effusion is present in 20% to 40% of hospitalized patients with pneumonia, and approximately 5% to 10% of these patients progress to empyema. The mortality rate accounts for 10% to 20%, despite systemic antimicrobial treatment, approximately 30% of them often requiring surgical treatment [6,7].

SAG, formerly known as *Streptococcus milleri*, consists of three organisms, including *S. anginosus*, *Streptococcus *​​​​​*intermedius*, and *Streptococcus constellatus*. SAG is a *Viridans streptococci* subgroup. They are nonmotile facultative anaerobes. These organisms are considered normal human flora in the oropharynx, gastrointestinal tract, and genitourinary tract [8]. However, these bacteria have been found to be pyogenic in some cases. SAG members exhibit specific virulence factors that are likely to be implicated in their ability to cause invasive pyogenic processes. They produce a toxin with a leukocidin-like effect, which gives them the ability to cause abscesses. In addition, SAG species possess the thrombin-like activity and the ability to bind to platelet-fibrin clots, protecting themselves from antibodies and immune effectors [3].

Members of SAG are often associated with pharyngitis, sinusitis, and endodontic infections [4]. However, this group can uniquely extend beyond fascial planes and interlobar fissures. Lung abscesses that develop from necrotizing pneumonia communicate with the airway, undergo auto-drainage, and usually only require long-term antibiotics. Nevertheless, due to the SAG’s ability to cross fascial planes, the lung abscess is often complicated by empyema due to its rupture into the pleural cavity [9]. Okada et al. [1] reported that a concomitant pleural effusion was encountered in 54.5% of patients with lung abscesses caused by SAG infections and that pleural effusions accompanying lung abscesses were more frequently observed in patients with SAG infections than in patients infected with other pathogens. They speculated that respiratory infections caused by SAG should be especially considered in patients with pleural effusion [1].

Diagnosis of SAG lung abscess is clinical, accompanied by CT imaging. Treatment starts with empiric antibiotic therapy and may be tailored to a specific pathogen if isolated. On many occasions, due to the initiation of empiric antibiotics, cultures can result negative. Also, it has been seen that SAG infection is commonly associated with concomitant anaerobic infection, increasing SAG virulence [10]. The typical treatment for these infections is ampicillin or vancomycin plus drainage of any concomitant abscess. In a review by Giuliano et al. [3], nearly all isolates of the *S. anginosus* group are susceptible to amoxicillin and penicillin, although a few penicillin-resistant infections have been reported [3,4]. Timely treatment for lung abscesses is essential to hinder infection dissemination and prevent the grave complications of overwhelming sepsis and multiorgan failure.

Empyema is a common complication of lung abscesses and pneumonia. Treatment includes antibiotics and complete drainage of the infected fluid. Tube thoracostomy is useful in treating early stage, minimally septated empyema, and it should be combined with close CT follow-up to confirm the adequacy of drainage. Persistence of any undrained fluid should prompt additional drains such as small-bore catheters, intrapleural instillation of fibrinolytic with DNase, or more aggressive management. Per guidelines, a pleural fluid pH <7.2, LDH >1,000 IU/L, glucose <40 mg/dL, or a loculated pleural effusion suggests that the pleural effusion is unlikely to resolve with antibiotics alone. Surgical approaches include video-assisted thoracoscopic surgery and open thoracotomy [7].

## Conclusions

Clinicians should consider SAG as a possible causative agent in lung abscesses and empyemas, especially when the infection crosses tissue planes. Although the SAG incidence is low, infections are linked with high morbidity and mortality due to their rapidly evolving course.

Necrotizing pneumonia is a severe complication of pneumonia. If it is accompanied by empyema, the complexity of management is further increased. Although initial operative versus nonoperative management in empyema is controversial, cultures and sensitivities are essential to successfully tailor the treatment in individual cases and increase the chances of a successful outcome. Radiographic loculations are among the most significant predictors of failure when treating empyema.
